# Amacrine cells differentially balance zebrafish color circuits in the central and peripheral retina

**DOI:** 10.1016/j.celrep.2023.112055

**Published:** 2023-02-07

**Authors:** Xinwei Wang, Paul A. Roberts, Takeshi Yoshimatsu, Leon Lagnado, Tom Baden

**Affiliations:** 1 School of Life Sciences, University of Sussex, Biology Road, Brighton BN1 9QG, UK; 2 Institute of Ophthalmic Research, University of Tübingen, Elfriede-Aulhorn-Strasse 7, 72076 Tübingen, Germany

## Abstract

The vertebrate inner retina is driven by photoreceptors whose outputs are already pre-processed; in zebrafish, outer retinal circuits split “color” from “grayscale” information across four cone-photoreceptor types. It remains unclear how the inner retina processes incoming spectral information while also combining cone signals to shape grayscale functions. We address this question by imaging the light-driven responses of amacrine cells (ACs) and bipolar cells (BCs) in larval zebrafish in the presence and pharmacological absence of inner retinal inhibition. We find that ACs enhance opponency in some bipolar cells while at the same time suppressing pre-existing opponency in others, so that, depending on the retinal region, the net change in the number of color-opponent units is essentially zero. To achieve this “dynamic balance,” ACs counteract intrinsic color opponency of BCs via the On channel. Consistent with these observations, Off-stratifying ACs are exclusively achromatic, while all color-opponent ACs stratify in the On sublamina.

## Graphical abstract

**Graphical abstract:**
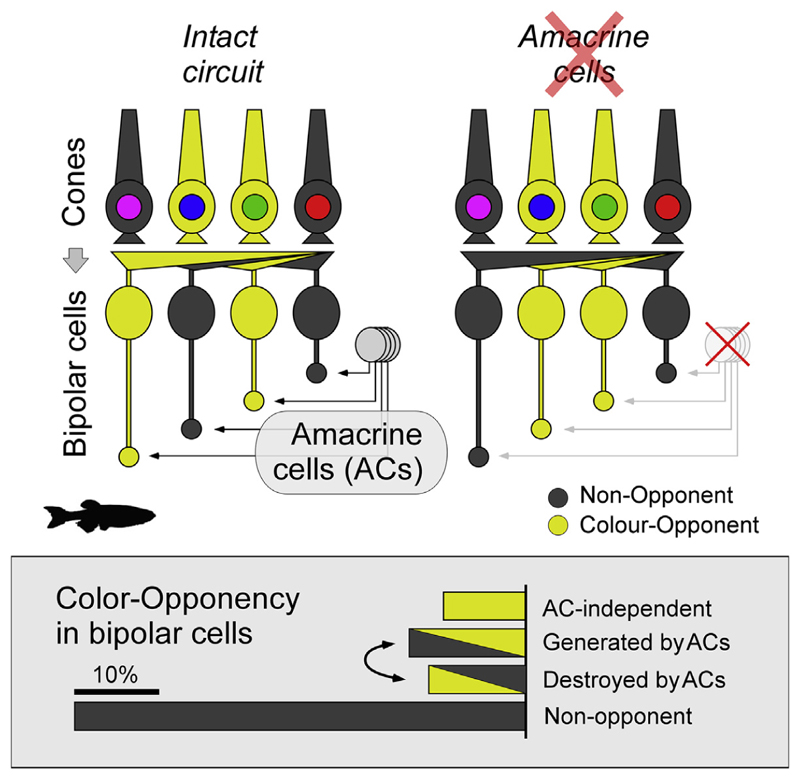

## Introduction

Animal eyes encode patterns of light along distinct axes of variation, such as space, time, and “color.”^1^ These axes combine signals from a shared population of photoreceptors, which poses a general question in neural circuit organization: how can a common set of inputs be processed so that specialization for one task does not simultaneously degrade function elsewhere?

One strategy is to split the neural signal down separate microcircuits that implement different processing tasks. Such parallel processing is fundamental to brain function,^2^ including in the vertebrate retina,^3,4^ where stimulus-response relationships become increasingly specific and diverse as the visual signal travels from photoreceptors via bipolar cells (BCs) to retinal ganglion cells (RGCs). Based on this architecture, progressive circuit specialization for distinct tasks can then take place in different populations of inner retinal neurons. A key question is: at which stage in the retinal circuit does specialization for each processing task occur? Specialization for one task may precede specialization for others. In teleost fish, for example, spectral coding in the outer retina^5^ precedes the extraction of key spatiotemporal features of the inner retina.^6^

In larval zebrafish, splitting of “color” and “brightness” signals begins in the outer retina, where the cone photoreceptors are modulated by horizontal cells (HCs) at the first synapse in vision.^7^ Red cones provide an output that signals brightness and is essentially color invariant, while green cones provide a primary color output that is brightness invariant. Blue and UV cones then provide secondary color and brightness channels, respectively. Such parallel representation^7–10^ of spectral information is beneficial for color vision,^11^ but how can it be maintained through the dense interconnectedness of inner retinal circuits?^12–14^ All of the various processing channels evident in the retinal output involve amacrine cells (ACs) in the inner retina that modify the visual signal by inhibition through GABA (γ-Aminobutyric acid) and glycinergic transmission alongside a variety of neuromodulators that can either inhibit or potentiate transmission.^14–17^ How do these operations on the visual signal in the inner retina adjust the results of spectral processing arriving from the outer retina? It is established that distinct types of BCs do represent the spectral inputs from each of the four cone signals in isolation, but along-side them are a plethora of other BCs that represent diverse cone mixtures, presumably specialized for other temporal and spatial processing tasks.^18,19^

ACs are the most diverse yet least understood class of neurons in the retina.^6,20,21^ In mice, transcriptomics analysis revealed 63 molecularly distinct types of ACs,^22^ while in zebrafish, more than 20 can be defined by anatomy.^12^ General roles of ACs include shaping of BC and RGC receptive field structures,^14,20^ modulating their dynamic range^23,24^ and adaptive properties to generate the functional diversity of the retinal output.^13,25,26^ Although the specific functions of most AC types across any species remain unknown, we might expect ACs to contribute to chromatic and achromatic signaling.^27–29^ To test whether this is the case, we surveyed light-driven signals of ACs *in vivo* across different eye regions of the zebrafish retina. Surprisingly, this revealed that, despite being highly diverse (for example, in terms of kinetics and polarity), ACs were mostly non-opponent and spectrally resembled linear combinations of UV- and red-cone signals, which in zebrafish are associated with grayscale processing.^7^ Next, we imaged BC light responses in the presence and absence of AC-mediated inhibition. This demonstrated that, while BC grayscale processing was profoundly altered across the entire retina, adjustments in color processing varied between different regions of the retina. While in the central retina ACs served to set up a new “UV-yellow” axis of color opponency, in the peripheral retina the population representation of spectral contrast^5,30^ was essentially invariant to the removal of inhibitory signals. However, this was not because opponency in individual BCs was invariant to AC block. On the contrary; ACs routinely abolished and generated spectral opponency at the level of individual BCs, but they did so in approximately equal measure, so that the net change across the population of BCs was essentially zero. To preserve the balance between different chromatic and achromatic channels, ACs act nearly exclusively through On circuits.

We conclude that distinct circuits serve to conserve the parsing of color information performed in the outer retina as the visual signal is transmitted to central and peripheral BCs. The inhibitory interactions within the inner retina that underly other visual processing tasks do not notably alter the population representation of color information.

## Results

### Surveying AC functions

**Figure 1:**
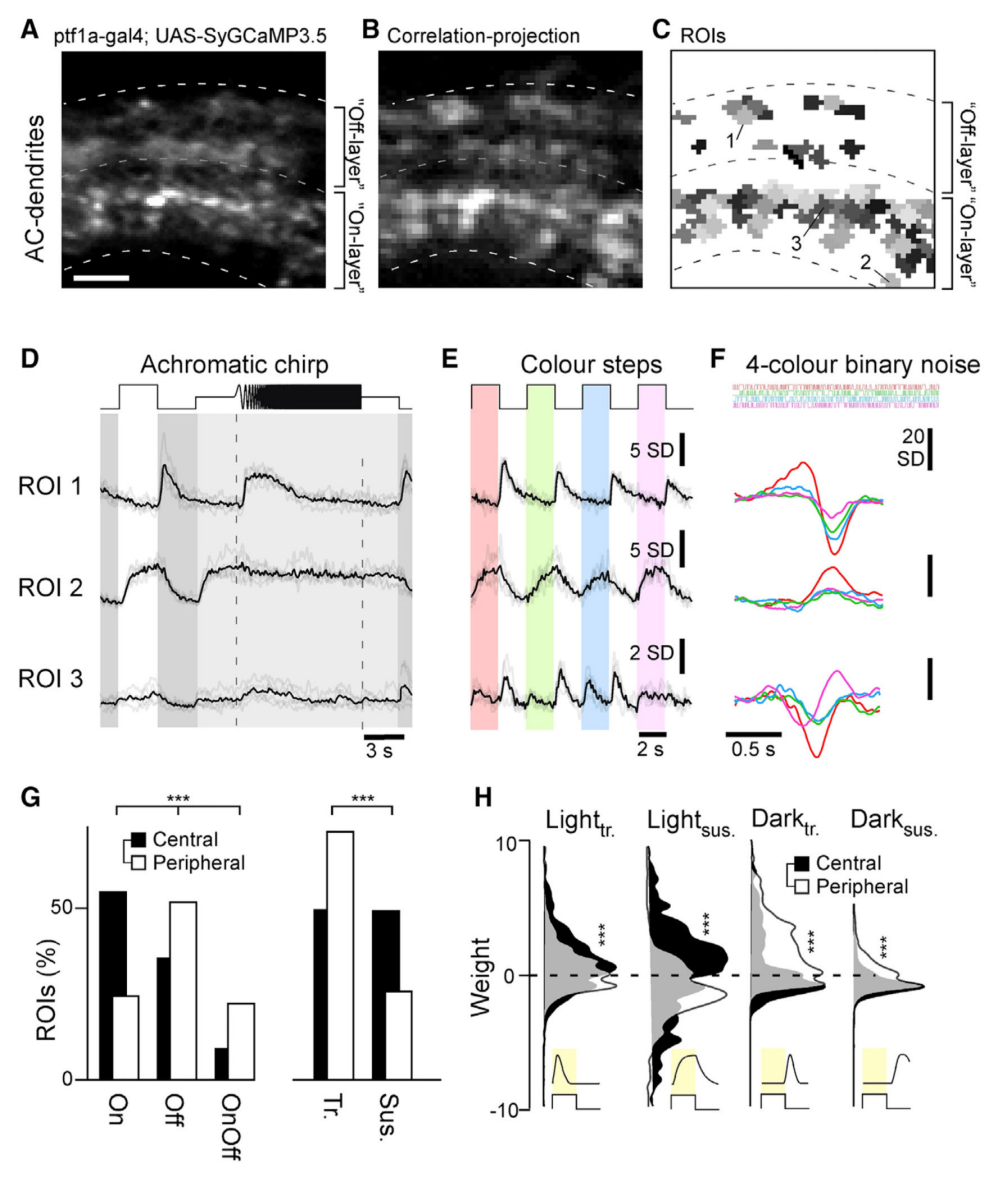
Recording AC functions *in vivo* (A–C) Example scan of syGCaMP3.5-expressing AC dendrites within the IPL, showing the scan average (A); a projection of local response correlation, indicating regions of high activity (B); and the correspondingly placed ROI map (C). Scale bar, 5 µm. (D–F) Example regions of interest (ROIs, cf. C), with mean trial averages (black) superimposed on individual repeats (gray) to the three light stimuli tested: an achromatic chirp stimulus (D), four spectrally distinct steps of light from dark (red, green, blue, UV, as indicated; STAR Methods) (E), and a spectral noise stimulus used to establish linear filters (kernels) at the same four wavelengths (F). (G) Distribution of ROIs from the central (black, n = 927 ROIs, 10 scans, 6 fish) and peripheral retina (white, n = 816 ROIs, 10 scans, 6 fish) by polarity (On/Off/On-Off) and kinetics (transient/sustained). Chi-square test, p < 0.001 for both datasets. (H) Distribution of kinetic component weights (STAR Methods; cf. Figure S1B) of central and peripheral ROIs. Wilcoxon rank-sum test, p < 0.001 for all four datasets.

**Figure 2:**
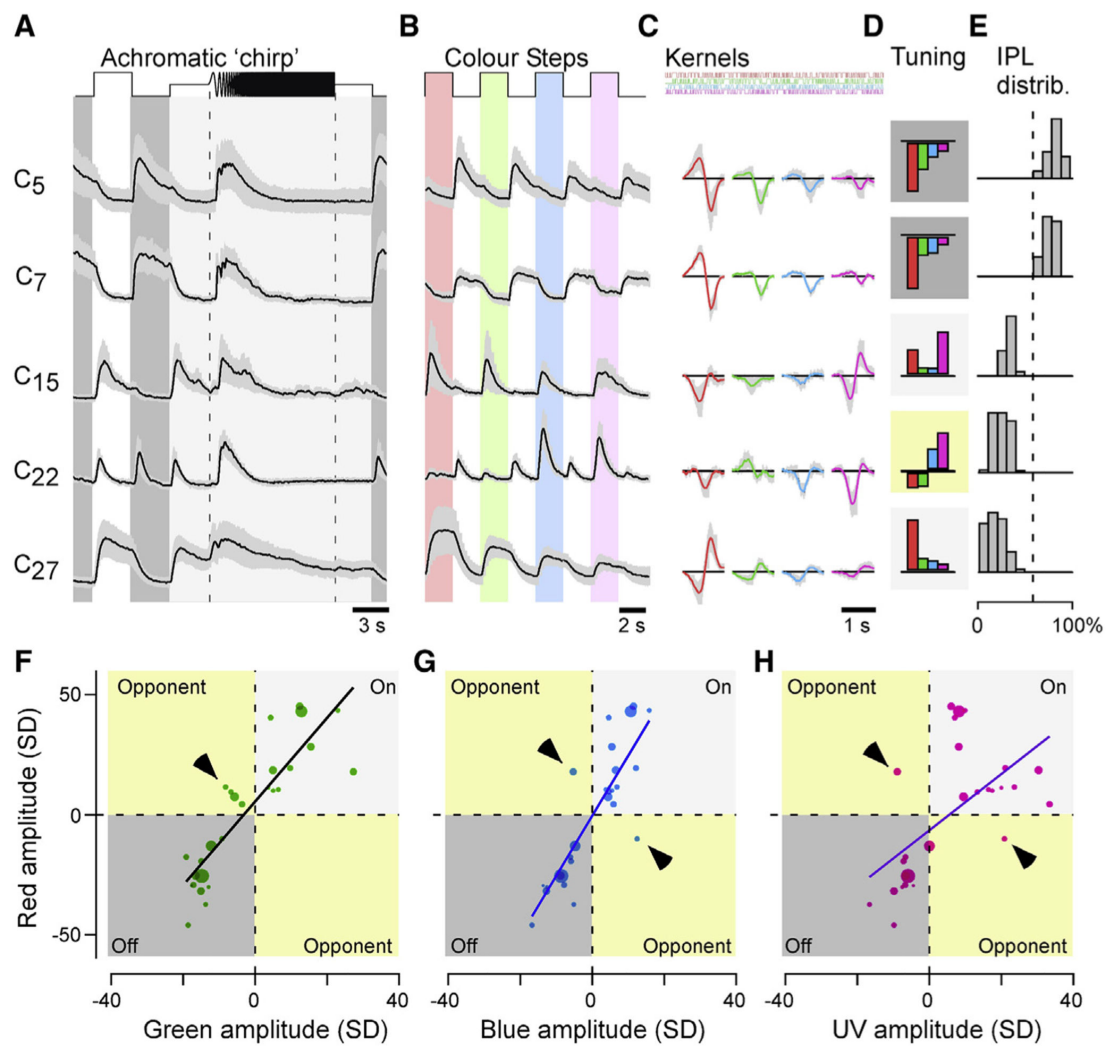
ACs are kinetically diverse but spectrally simple (A–E) Example cluster means ± SD in response to the white chirp (A) and color-step stimuli (B), spectral kernels (C) corresponding to mean kernel amplitudes (D), and each cluster’s distribution across the IPL (E). Shadings in (D) annotate cluster polarity (dark, Off; light, On; yellow, color opponent). (F–H) Relationship of each clusters’ kernel amplitudes (n = 27 clusters) in red (y axis) plotted against the corresponding amplitudes in green (F), blue (G), and UV (H). Note that most points scatter across the two non-opponent quadrants of the plots, with exceptions highlighted by arrowheads. Dot size indicates the number of ROIs in a cluster. Non-weighted line fits are superimposed for illustration.

**Figure 3:**
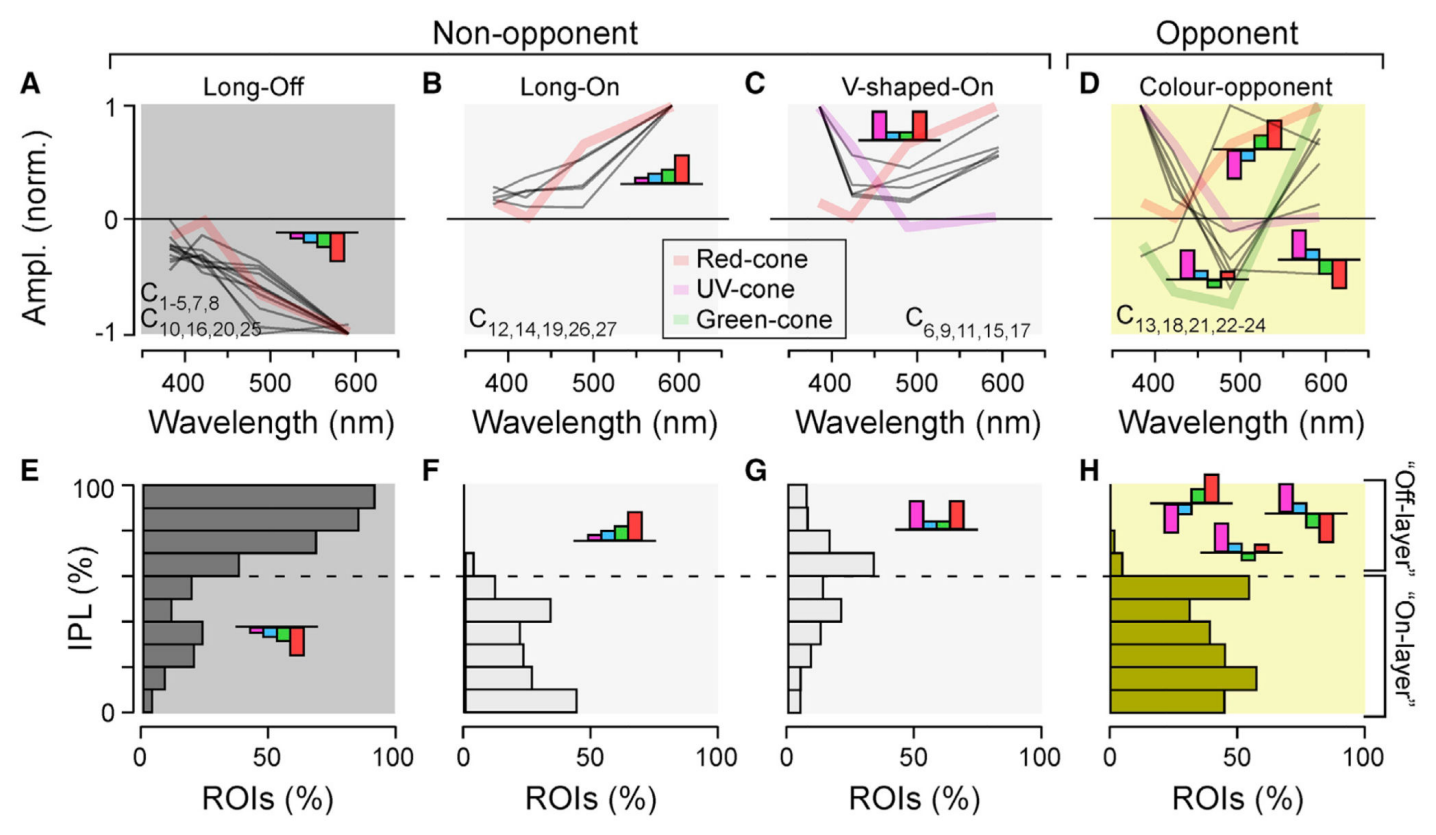
ACs are kinetically diverse but spectrally simple (A–D) Spectral tuning functions of all AC clusters (gray lines), allocated to one of four groups as shown: long-wavelength-biased Off (A) and On (B), V-shaped On (C), and color opponent (D). Plotted behind the AC spectral tunings are reduced tuning functions of selected cones (cf. Figures S5B and S5C) to illustrate qualitative spectral matches between cones and AC clusters. Clusters contributing to each group are listed in each panel. (E–H) Corresponding IPL distribution of AC ROIs allocated to each of the four spectral groups, as indicated.

To investigate how inhibitory microcircuits in the inner retina contribute to processing, we began by recording how the population of ACs in larval zebrafish encode grayscale and color information across the inner plexiform layer (IPL) and across different parts of the eye. For this, we used *in vivo* 2-photon imaging of SyGCaMP3.5 expressed under the ptf1a promoter, which targets the vast majority of ACs in zebrafish^25,31^ (Figures 1A–1F). We recorded dendritic calcium responses of ACs to a battery of wide-field light stimuli testing basic visual processing tasks (STAR Methods): (1) an achromatic (“white”) step of light (3 s On, 3 s Off, 100% contrast), testing response polarity and kinetics (Figure 1D); (2) a frequency-modulated chirp centered at 50% contrast, testing frequency response (Figure 1D); (3) steps of light (2 s On, 2 s Off, 100% contrast) at four different wavelengths (red, 592 nm; green, 487 nm; blue, 420 nm; UV, 382 nm), testing spectral sensitivity (Figure 1E); and (4) “tetra-chromatic binary noise” (5 min, 6.4 Hz, 100% contrast), which allowed us to extract four “spectral sensitivity kernels” per terminal to probe for spectral opponency (Figure 1F; STAR Methods). This set of stimuli was chosen to facilitate comparison with previous work^7,13,19,32,33^ and to test a wide range of achromatic and spectral processing tasks within a limited recording time.

We recorded from two regions of the eye: (1) the central “fovea-like” *area temporalis*, which surveys upper frontal visual space to support binocular vision, including prey capture,^19,34–38^ and (2) the peripheral nasal retina, which surveys the outward horizon (n = 10 scans each). Regions of interest (ROIs) were detected at all IPL depths, with most AC responses occurring in two major bands, toward the respective centers of the traditional On and Off layers (Figure S1A). In total, we recorded from 927 ROIs in the central retina and 816 ROIs from the peripheral retina.

AC processes exhibited diverse responses across the tested battery of stimuli (Figures 1D–1F). ROI 1, for example, consistently exhibited transient Off responses to the white chirp stimulus (Figure 1D) and when probed with color flashes (Figure 1E), while ROI 2 exhibited sustained On responses. These different behaviors were also captured by the spectral kernels (Figure 1F), which additionally highlighted an overall preference for long-over short-wavelength stimulation in both cases. In contrast, ROI 3 responded poorly to the “white” chirp but exhibited a variety of On and Off responses to different-wavelength color flashes. In this case, the spectral kernels indicated a gradual shift from long-wavelength Off dominance to short-wavelength On dominance, identifying this ROI as color opponent.

### Variations in AC function in the central and peripheral retina

The larval zebrafish retina is structurally and functionally asymmetrical to acknowledge statistical asymmetries in the natural visual world as well as species-specific visual demands.^19,34,35,39,40^ For example, the central retina comprises a high density of UV On circuits to support prey capture,^19,33,34,41^ while the nasal and dorsal parts of the eye disproportionately invest in color-opponent circuits^18,19^ to survey the color-rich horizon and lower visual field, respectively. To evaluate how ACs might contribute to regional differences in the distribution of functional microcircuits, we began by analyzing responses to the white step of light, fitting these to a linear kinetic model as described in recent work.^18^ Briefly, the model used four kinetic templates to capture the dominant response waveforms across our datasets: light transient, light sustained, dark transient, and dark sustained (Figure S1B; STAR Methods). This procedure simplified complex responses into four components and their corresponding weights. For example, ROI 2 (Figure 1D) exhibited a relatively slow On response that was readily captured by a positively weighted Light-sustained component alone (Figure S1B, bottom). The Off response of ROI 1 was kinetically more complex. Capturing this compound waveform required combined use of a small, negatively weighted, light-sustained component alongside a large, positively weighted, Dark-transient component (Figure S1B, top). The same approach served to fit all AC achromatic step responses, consistently capturing more than 93% of the variance across response means.

The extracted component weights allowed us to quantitatively compare the distribution and types of responses across the central and peripheral retina based on two fundamental properties: polarity (On, Off, On-Off) and kinetics (transient, sustained; Figure 1G; STAR Methods). The central retina was On dominated (54.5% of ROIs compared with 35.6% Off and 9.4% On-Off ROIs), while the peripheral retina was Off dominated (51.7% of ROIs compared with 24.5% and 22.4% of On and On-Off ROIs, respectively; 1.4% non-responders). Moreover, while the central retina comprised approximately equal fractions of transient (49.8%) and sustained (49.5%) responses, the peripheral retina was heavily dominated by transient (72.6%) responses. Further insights emerged by comparing the distributions of kinetic component weights across retinal regions (Figure 1H). Central ACs were dominated by large positive and negative light-sustained weights alongside a smaller contribution from positive light-transient weights. In contrast, peripheral ACs drew on a more varied distribution of kinetic components and with a particular emphasis on the use of positive Dark-transient weights. ACs from the two retinal regions therefore exhibited a variety of responses to a simple achromatic step of light.

We next sorted ACs into response types based on the full range of stimuli by using a “mixture of Gaussians” model to jointly cluster all ROIs irrespective of IPL position or eye region (STAR Methods). This returned 27 clusters that further highlighted the striking functional differences in AC functions across the eye (Example clusters are shown in Figures 2A–2E and a complete overview in Figures S2 and S3). Regional information was not used to drive the clustering (see Figure S4 for an alternative clustering by eye region), but 20 of the 27 clusters nonetheless comprised ROIs exclusively from either the central or peripheral retina. The remaining seven clusters comprised various mixtures of ROIs from both regions.

### ACs are kinetically diverse but spectrally simple

Using the output of the joint clustering procedure, we compared how ACs encode grayscale and color information. As we detail below, this revealed that ACs could be divided into two main spectral groups: a majority of kinetically diverse but spectrally simple achromatic ACs and a minority of color-opponent ACs with complex spectral responses, which stratified exclusively in the On layer.

21 of the 27 clusters responded similarly to the white and colored steps of light (Figures 2A and 2B), a behavior that was also captured in the spectral kernels (Figures 2C and 2D). For example, cluster C_5_ exhibited transient Off responses to steps of light at any wavelength, while cluster C_7_ consistently displayed sustained Off responses. Correspondingly, each of the four spectral kernels also indicated Off behavior (Figure 2C), rendering these clusters non-opponent. Similarly, the kinetically distinct On clusters C_15_ and C_27_ were also non-opponent. The remaining six clusters were more complex. For example, C_22_ displayed transient On-Off responses at all tested wavelengths but with a notable Off dominance during long-wavelength stimulation and On dominance during short-wavelength stimulation, rendering this cluster color-opponent overall. Such wave-length-dependent rebalancing of On versus Off amplitudes, rather than a “classic” full polarity reversal as observed in cones^7^ and BCs,^18,19^ also rendered the remaining five clusters weakly opponent overall.

The dominance of non-opponent responses among AC clusters was further illustrated by comparison of kernel amplitudes across different wavelengths (Figures 2F–2H). For example, pair-wise comparison of red- versus green-kernel amplitudes highlighted that most clusters exhibited same-sign behavior at both wavelengths (Figure 2F). Only a minority were red-green opponent (arrowhead), and these clusters notably also exhibited the lowest kernel amplitudes overall. Qualitatively similar behavior was observed when comparing red-blue and red-UV wave-lengths (Figures 2G and 2H).

The 27 AC clusters could be sorted into four spectral groups (STAR Methods): Three large non-opponent groups (Off-long-biased, C_1-5,7,8,10,16,20,25_, On-long-biased, C_12,14,19,26,27_; “V-shaped,” C_6,9,11,15,17_) and one opponent (comprised of mid-wave-length opponent clusters C_21,22_ and “green opponent” clusters C_13,18,23,24_; Figures 3A–3D). This simplification illustrated their remarkable spectral homogeneity and further facilitated summarizing their distributions across the IPL (Figures 3E–3H); all 11 Off clusters as well as 5 of the 10 On clusters followed a common, long-wavelength-biased and non-opponent spectral tuning function (Figures 3A and 3B). Off-long-biased ROIs were mostly, but not exclusively, located in the IPL’s traditional Off layer (Figure 3E), while On-long-biased ROIs were only found in the On layer (Figure 3F).

All remaining non-opponent On clusters fell into a third group that was spectrally “V shaped” (Figure 3C), with ROIs exhibiting an incomplete bias to the center of the IPL. This last group was overwhelmingly comprised of ROIs from the central retina (Figure S5A), which is known to be heavily UV dominated.^18,19,33,34^ The spectral tuning of all 21 non-opponent ACs clusters was readily explained by inputs from red and UV cones (Figures S5B–S5E), which are associated with achromatic processing.^7^ Strikingly, essentially all ROIs in the Off layer and approximately half in the On layer were of this non-opponent population. The remaining ROIs in the On layer consisted of six AC clusters that were kinetically and spectrally complex and weakly but consistently color opponent (central, 28.4%; peripheral, 29.2%; Figures 3D and 3H). Of these, green-opponent clusters predominated in both regions, along-side functionally opposite mid-wavelength opponent clusters (central C_22_, long−/short+; peripheral C_21_, long+/short−). The spectral behavior of color-opponent clusters could not generally be explained without additional inputs from the opponent^7^ green cones, which are associated with color processing (Figures S5B–S5E).

We next tested how these different distributions of AC functions across the eye and IPL might be linked to spectral and temporal processing in BCs.

### BC signaling in the presence and absence of inhibition from ACs

**Figure 4:**
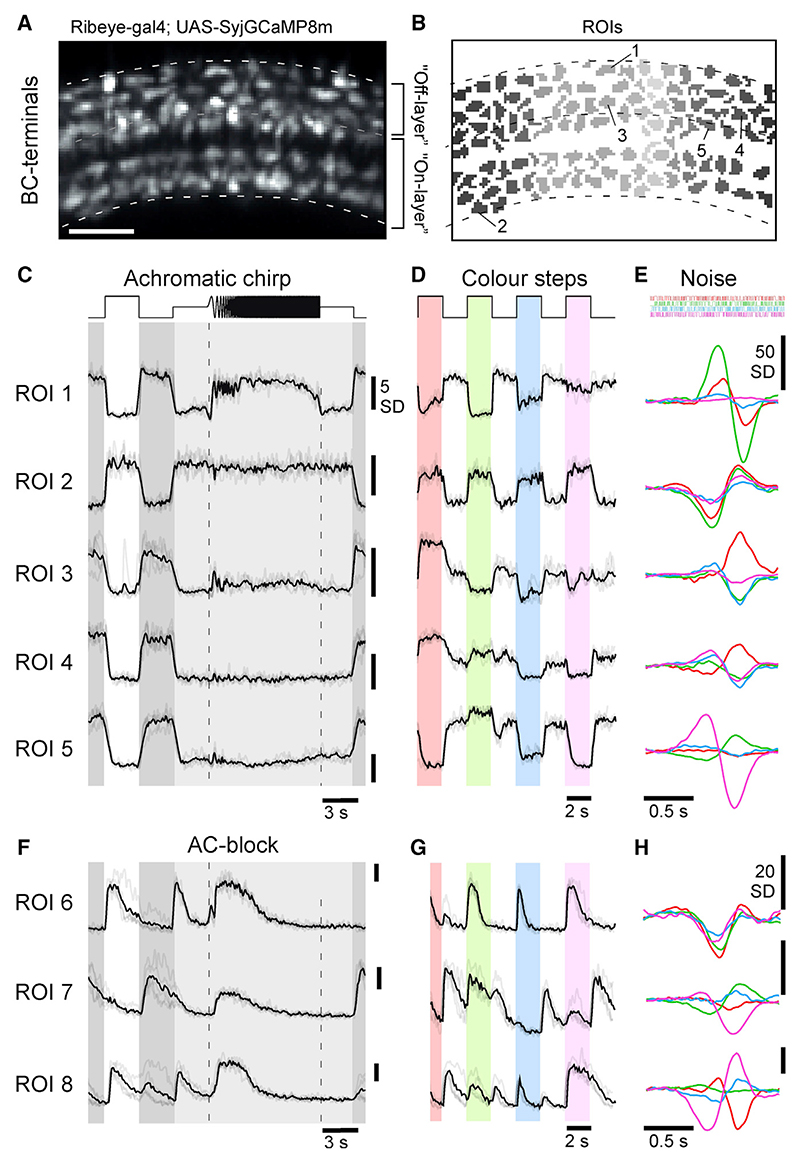
Effects of AC blockage on BCs (A and B) Example scan field (A) and ROI mask (B) of a typical scan from BC terminals expressing SyjGCaMP8m,^44,45^ with approximate IPL boundaries and On/Off layer separation indicated. Scale bar, 5 µm. (C–E) Example ROIs from (A) and (B) as indicated, responding to the white chirp (C) and color steps (D), alongside mean spectral kernels recovered from 4-color noise stimulation (E). (F–H) As (C–E), respectively, but for three different example terminals that were recorded after pharmacological injections of GABAzine, TPMPA, and strychnine to block inhibitory inputs from ACs. Note that responses are generally larger and more transient compared with control conditions, but diverse spectral opponencies persist. Scale bars: 5 SD (C, D, F, and G), 50 SD (E), and 20 SD (H).

To investigate the effects of AC-mediated inhibition on the visual signal transmitted through the inner retina, we combined pharmacology with *in vivo* 2-photon (2P) imaging of BC synaptic terminals expressing the calcium biosensor SyjGCaMP8m^18,42–44^ (Figures 4A–4F; STAR Methods). In each experiment, we first scanned ~10° eye regions comprising typically 100–120 individual BC terminals (Figures 4A and 4B) and presented the same battery of stimuli used previously to characterize ACs (Figures 4C–4E). Next, we injected a cocktail of gabazine, TPMPA (1,2,5,6-Tetrahydropyridin-4-yl)methylphosphinic acid), and strychnine into the eye to pharmacologically block GABA_A_, GABA_C_, and glycine receptors, respectively^26^ (STAR Methods), which represent the major known sources of AC-mediated inhibition in the inner retina.^15^ We then imaged the inner retina a second time (e.g., Figures 4F–4H) to compare the functions of BC terminals in the presence or pharmacological absence of AC-mediated inhibition.

**Figure 5:**
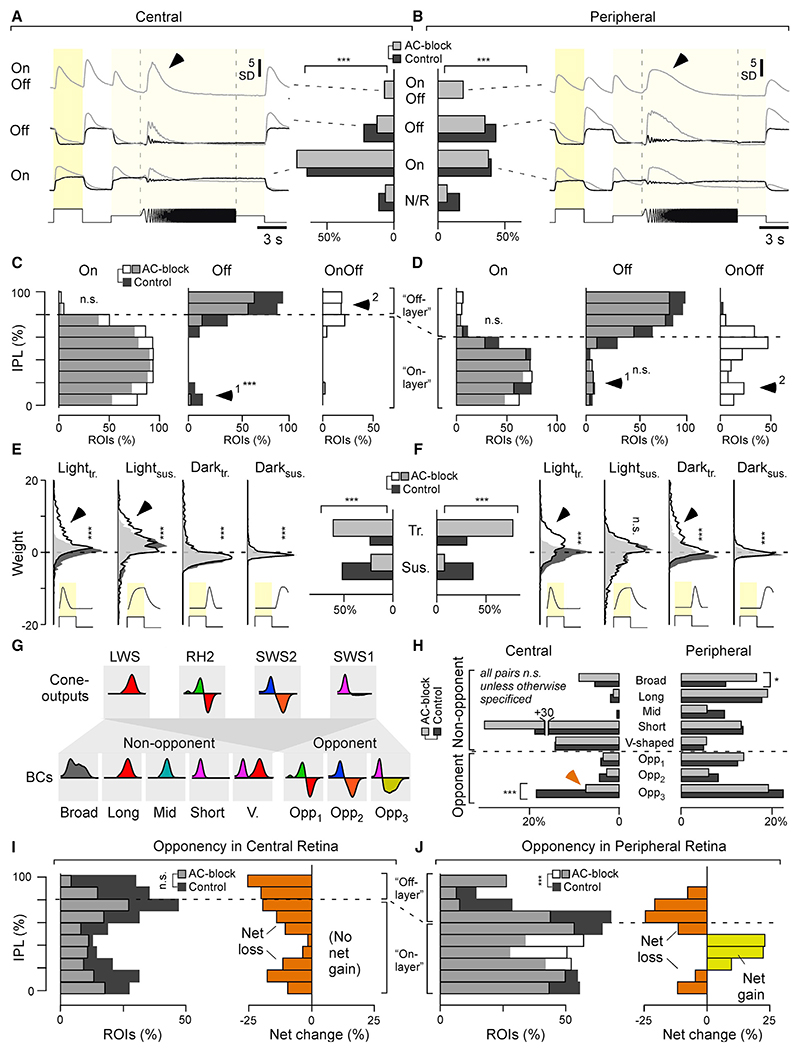
ACs differentially modulate grayscale processing in BCs across the eye (A and B) Bar plots: distribution of BC ROIs recorded in the central (A) and peripheral retina (B) by response polarity (non-responsive, On, Off, On-Off) before (control, dark gray) and after blocking inhibitory inputs (AC block, light gray), as indicated (chi-square tests for the bar plots, p < 0.001 for both datasets), and the corresponding mean response waveform to the white chirp stimulus (cf. Figure 4C) of all terminals in a given category. Arrowheads highlight distinct temporal dynamics during temporal flicker across the regions. Central: n = 1,492 ROIs under the control condition and 1,566 ROIs under the AC block condition from 13 scans in 13 fishes; peripheral: n = 1,526 ROIs under the control condition and 1,639 ROIs under the AC block condition from 13 scans in 13 fishes. (C and D) Distribution of On, Off, and On-Off BC terminals across the IPL, as indicated, with control data shown in dark gray and AC block data superimposed semi-transparently in white, so that overlapping bars appear in light gray. Note that the On/Off boundary differs between the central (C) and peripheral retina (D; see also Bartel et al.,^18^ Zimmermann et al.,^19^ and Zhou et al.^33^). Arrowheads highlight key differences between central and peripheral histogram pairs. Two-sample Kolmogorov-Smirnov test; central: On, p = 0.77; Off, p < 0.001; peripheral: On, p = 0.30; Off, p = 0.11. Because of the absence of data points under control conditions, no statistical tests were used to test changes for On-Off distributions. (E and F), Distribution of kinetic component weights used to fit BCs’ white step responses (cf. Figure S1B; STAR Methods) across the datasets and conditions as indicated (vertical histograms) and distribution into overall transient and sustained response types (bar plots). Arrowheads highlight the major differences across control and AC block conditions in each case. Wilcoxon rank-sum test for the distribution plots, p = 0.93 for the peripheral light_sus_ group, p < 0.001 for all other groups; chi-square test for the bar plots, p < 0.001 for both datasets. (G) Illustration of how cone input could build the different spectral response types in BCs. Insets indicate approximate spectral tuning functions (amplitude y versus wavelength x). The top row shows the four cones,^7^ and the bottom row shows BCs,^18,19^ divided into five non-opponent and three opponent categories. (H) Percentages of ROIs per spectral category (cf. G) based on BC kernels (cf. Figure 4E) for central (left) and peripheral datasets (right). Note that the distribution across spectral categories differs strongly by retinal region, but within a region, most spectral categories are approximately stable between control (dark gray) and AC block conditions (light gray). The main exception to this observation is indicated by the arrow. Wilcoxon rank-sum tests, central: p > 0.05 for the first 7 groups, p < 0.001 for “Opp_3_” group; peripheral: p = 0.015 for the “Broad” group, p > 0.05 for all others. (I and J) Left: IPL distributions (I, central retina; J, peripheral retina) of all ROIs classed as color opponent during the control condition (dark gray) and following AC block (white, so that their overlay is light gray). Right: difference between control and AC block in each case, with net loss of opponency shown in orange and net gain in yellow. The full datasets leading to the summaries in (H–J) are shown in Figures S7B–S7E. Two-sample Kolmogorov-Smirnov test for changes in IPL distributions of opponent terminals; central, p = 0.39; peripheral, p < 0.001.

The efficacy with which this manipulation blocked inhibition in the inner retina was evidenced by the increase in the gain of responses in BC synapses (Figure 5; Video S1) and the increased synchronicity^13^ of these responses (Figures S6A and S6B). We also evaluated the effect of blocking inhibitory receptors on outer retinal function, where horizontal cells spectrally retune the cone output.^7^ Using existing cone-type-specific SyGCaMP6f lines,^7^ we confirmed that the cones’ spectral tunings were invariant to application of the drug cocktail (Figures S6C–S6N).

In line with previous work,^18,19,46^ BCs displayed a broad range of response properties under control conditions, which included On and Off cells with diverse temporal and spectral tunings. For example, in a scan from the peripheral retina (Figures 4A and 4B), ROIs 1 and 2 displayed largely achromatic Off and On responses, respectively, while ROIs 3–5 exemplified different forms of color opponency (Figures 4C–4E; Video S2).

A substantial degree of functional diversity in BC responses was also observed following pharmacological AC block, including the continued presence of numerous color-opponent responses (Figures 4F–4H). However, the nature and distribution of these disinhibited responses were profoundly altered compared with control conditions and in a manner that system-atically differed between the central and peripheral retina, as we describe below.

### Changes in grayscale processing caused by blocking inhibition

The most general effects of blocking inhibition from ACs were to make responses in BCs larger and more transient, and this occurred across retinal regions and for terminals of all polarities (Figure 5). By fitting step responses to the same four kinetic components used previously to fit ACs, we could account for more than 94% of the variance across BCs. Using the kinetic weights, we automatically classified each BC response as unresponsive, On, Off, or On-Off and computed the average chirp-response traces for the latter three categories per retinal region and condition (Figures 5A and 5B). In the central and peripheral retina, blocking ACs reduced the number of Off and unresponsive terminals and unmasked the presence of “intrinsically On-Off” terminals. Following block of AC inputs, On-Off terminals were also observed in response to colored stimuli, most notably to red and UV (Figure S7A), but they were never observed under control conditions.

Other effects of blocking inhibition were dependent on retinal region. Unmasking of On-Off responses, for example, was much more common in the peripheral compared with the central retina. Moreover, on average, peripheral BCs of all polarities followed the frequency-accelerating part of the chirp for longer compared with central BCs, suggesting regional differences in the modulation of temporal processing. Overall, while blocking ACs mainly accentuated the pre-existing On bias of the central retina, the same manipulation yielded a more complex re-distribution of response properties in the periphery.

To analyze how these changes in BC function were distributed across the IPL, we segregated terminals into 10 strata and computed histograms summarizing the relative depth distributions of On, Off, and On-Off terminals in each region and condition (Figures 5C and 5D). Blocking inhibition from ACs had distinct effects in the central and peripheral retina. In the central retina, ectopic Off responses in the On layer were abolished (Figure 5C, arrowhead), but these were not affected in the peripheral retina (Figure 5D, arrow-head 1). In the central retina, blocking inhibition also generated mixed On-Off responses in the Off layer (Figure 5C, arrowhead 2), while in the peripheral retina On-Off response appeared throughout the IPL (Figure 5D, arrowhead 2). These results demonstrate that ACs do not simply regulate the gain and kinetics of the output from BCs but also the polarity. In the absence of inhibition, 19.3% of BC terminals in the peripheral retina signal On and Off transitions, and these were predominantly in the On layer. This previously unrecognized function of ACs, regulating the polarity of synaptic activity in BCs, was less prominent in the central retina, where it was only evident in 7.6% of terminals, and these were predominantly in the Off layer.

Regional differences in the way that ACs interact with BCs were also evident in the temporal domain (Figures 5E and 5F). Responses tended to become more transient after blocking inhibition, but this effect was much stronger in the periphery, where it involved an accentuation of light- and dark-transient response components (Figure 5F, arrowheads). In contrast, kinetic changes in the central retina were more moderate and restricted to light-transient and light-sustained components (Figure 5E, arrowheads).

These results demonstrate that ACs interact with BCs in a highly regional manner. In the central retina, ACs regulate the gain and speed of responses, suggesting that here, ACs primarily serve as a gain-control system.^47^ But in the peripheral retina, ACs also regulate the segregation of On and Off signals in the On layer. Below we ask how these functional reorganizations of the inner retina impact spectral processing.

### Changes in color processing caused by blocking inhibition

To distinguish changes in wavelength from changes in intensity, circuits for color vision contrast signals of different photoreceptor systems.^5,30^ The resultant color-opponent neurons can be considered the fundamental “currency” of color vision. In zebrafish, BCs represent three types of spectral opponency (Figure 5G): long (“red-green”), mid (“orange-blue”), and short (“UV-yellow”), with spectral zero crossings at ~ 523, ~ 483, and ~450 nm, respectively. ^18,19^ Of these, long- and mid-wavelength opponency is already encoded at the level of green and blue cones, respectively,^7^ implying that ACs are not necessary to establish these channels in BCs. In contrast, short-wavelength opponency (“UV-yellow”) is only weakly represented in UV cones^7^ but dominant amongst BCs.^18^ The expectation, therefore, is that short-wavelength opponency requires the activity of ACs. This expectation was confirmed in the case of BCs in the central retina but not the periphery.

Opponency in BCs was assessed from spectral kernels computed from color-noise responses, as used for classifying ACs (Figures 3A–3D). However, because BCs^18,19^ are more spectrally diverse than ACs (Figures 1–3), we classified each into one of eight (rather than four) groups: five non-opponent groups (broad, long-biased, mid-biased, short-biased, V-shaped) and three opponent groups (long-opponent, Opp_1_; mid-opponent, Opp_2_; short-opponent, Opp_3_; Figures 5G and 5H; for full classification, see Figures S7B–S7E). This group allocation confirmed previous results^18,19^ showing that, under control conditions, the distribution of spectral response types strongly differed across the two regions. While in the central retina, spectral groups that required strong UV input accounted for the vast majority of responses, the diversity of spectral response types was much more evenly distributed in the peripheral retina, including greater numbers of color-opponent neurons (peripheral, 43.3%; central, 27.9%). Strikingly, in both retinal regions, these spectral distributions remained largely unchanged following AC block; no major spectral response group disappeared altogether (Figures 5H and S7B–S7E), and the abundance of many spectral groups was unchanged. For example, the numbers of V-shaped non-opponent responses appeared to be entirely unaffected in both retinal regions, while most color-opponent groups exhibited only marginal changes.

The only significant effect of blocking inhibition on color opponency that we could detect was a loss of short-opponent responses in the central retina in favor of a corresponding gain in short-biased, non-opponent responses (Figure 5H, arrow). This change was not observed in the peripheral retina, where all three opponent groups persisted throughout the pharmaco-logical manipulation.

Having established that the short-opponent interactions between ACs and BCs were specific for eye region, we looked more closely at inhibitory circuits at different locations in the IPL. For simplicity, the distribution of color opponency was assessed by summing the three color-opponent groups into a single distribution per experimental condition (Figures 5I and 5J; individual distributions are shown in Figures S7C and S7E). The color-opponent responses appearing after block of inhibition were short and long wavelength opponent (Figure S7E, arrowheads).

This finer analysis revealed that, despite the overall numerical conservation of all three opponencies in the peripheral retina following block of inhibition (Figure 5H), this was made up of a loss of opponency in the Off layer and a gain in the On layer (Figure 5J).

### ACs create and mask color opponency in individual BCs

To investigate how blocking inhibition caused a redistribution of color opponency in BCs, we tracked the same terminals across recordings. This approach was not possible when expression of SyGCaMP was driven in all BCs because the high density of terminals in the IPL made it difficult to reliably identify the same terminal before and after pharmacological manipulation. We therefore performed a new set of experiments using a different transgenic line where BCs expressed SyGCaMP3.5 sparsely (Figure S8A). This strategy allowed us to record from 20–30 individual terminals at a time, of which 40%–60% could be reliably matched across control conditions and after blocking inhibition (Figures S8A–S8D). We sampled 182 terminals from 14 fish covering the entire depth of the IPL. Because labeling was sparse, we combined all paired data into a single eye-wide dataset. We also recorded an equivalent but independent sham control dataset (n = 6 scans, n = 144 paired terminals), where we replaced the drug cocktail used to block inhibition with an equivalent volume of non-pharmacologically active vehicle. Sham injections had no significant effect on the functions we analyzed (Figure S9; STAR Methods).

**Figure 6:**
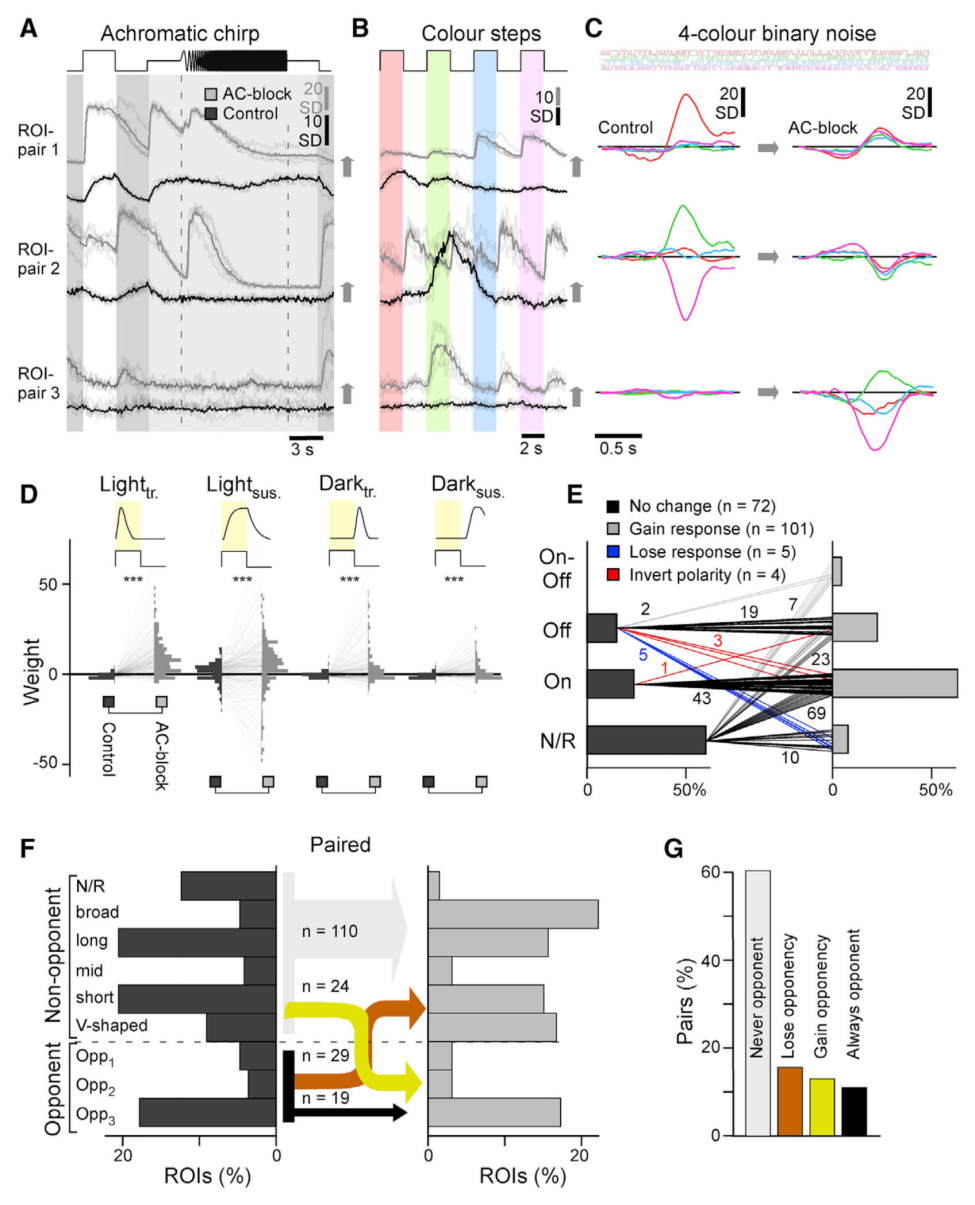
Paired recordings (A–C) Example ROI pairs as indicated in Figures S8A–S8D, illustrating frequently observed effects of AC block on single terminals when probed with the same set of stimuli used to classify all ACs (Figure 1) and BCs (Figure 4). Chirp and color-step traces (E and F) are vertically offset between the two conditions for clarity. (D) As Figures 5E and 5F but here for paired data (n = 182 pairs, from 13 scans, 13 fishes); pairwise weight distributions for the four kinetic components under the control (left) and AC block condition (right), as indicated. (E) Paired BCs automatically sorted by polarity under control (left) and AC block conditions (right). Lines represent the pairs, with numbers of BCs indicated. Chi-square test, p < 0.001. (F) As Figure 5H, allocation of BCs into spectral groups, here shown for a paired dataset, which allowed connecting spectral groups under the control condition (left) and following AC block (right). For simplicity, connections are collapsed into those where there is no change in color opponency (gray, black), where color opponency is lost upon AC block (orange), and where opponency is gained (yellow), with numbers of BC pairs corresponding to each connection indicated. Note that non-responsive terminals are added as one extra non-opponent category, which resulted from the generally lower signal-to-noise ratio in SyG-CaMP3.5 recordings compared with the previously used SyjGCaMP8m. (G) Summary of changes to color opponency as shown in (F).

As expected, blocking inhibition generally disinhibited BCs, resulting in less selective, more transient, and larger amplitude responses (Figures 6A–6E).

As in our population dataset, changes in spectral processing were diverse. In ROI pair 1, responses to color steps (Figure 6B) were red biased during control conditions but responded to all four wavelengths after blocking inhibition, and this spectral broadening was also observed at the level of the kernels (Figure 6C). It appears that, in this case, ACs were masking an intrinsic short-wavelength response to set up a long-wave-length biased BC. However, the effects on ROI pairs 2 and 3 were functionally opposite; ROI pair 2 exhibited a green-UV color-opponent response during control conditions, which was abolished following AC block, while, vice versa, ROI pair 3 exhibited a weak non-opponent response during control conditions but green-UV opponency upon AC block. Accordingly, in ROI pair 2, ACs were responsible for setting up BC opponency, while in ROI pair 3, ACs masked an intrinsic form of BC opponency.

**Figure 7:**
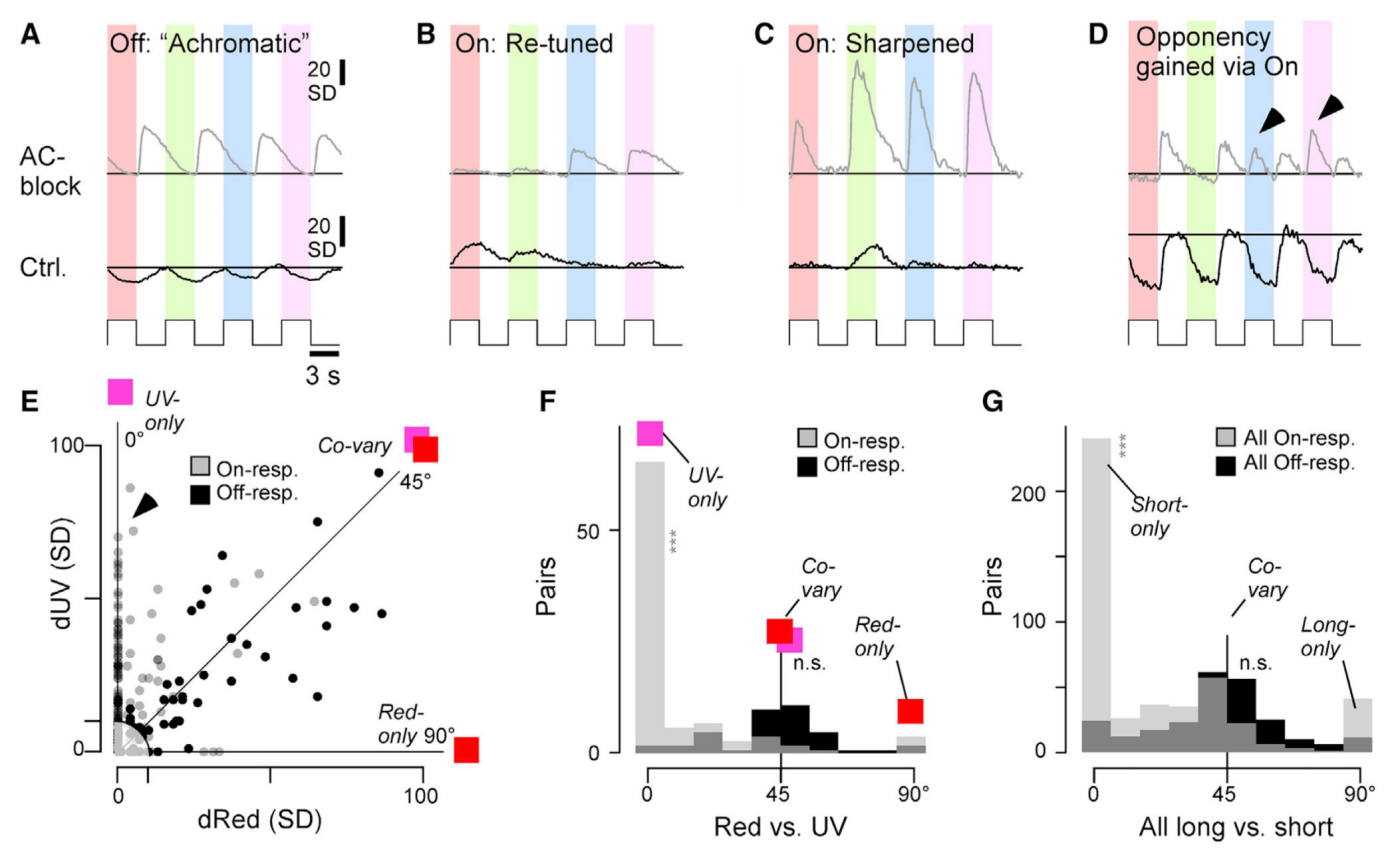
Selective spectral modulation of the On pathway (A–D) Selected example BC color-step responses (trial averages) during the control condition (bottom) and following AC block (top, paired data), illustrating a range of “typical” results. Generally, changes in Off responses tended to be similar across all tested wavelengths (A). In contrast, changes in On responses tended to be more spectrally diverse and often effected a change in overall spectral tuning (B and C), including in spectral opponency (D). Arrowheads in (D) highlight wavelength-specific switches in the On channel that led to a change in color opponency. (E–G) Co-variation of absolute response amplitudes changes (in SD) after AC blockage, compared across different pairs of wavelengths (paired data). (E) shows an individual scatterplot for red versus UV. On and Off responses are plotted separately, as indicated. Correspondingly, an angular histogram (F) was computed from this scatterplot, where 45° indicates co-variation, while peaks around 0° and 90° indicate that one of the two compared wavelength responses changes independently of the other. Data points with an Euclidean distance of less than 10 from the origin were excluded from further analysis (shaded area in E). (F) shows the individual red-UV comparison, while (G) shows the sum of all six possible color combinations (cf. Figure S10). Note that Off responses tended to co-vary (peak at 45°), while On responses exhibited a more diverse distribution, which included peaks at 0°, 45°, and 90°. Wilcoxon signed-rank test for a given color combination; tests were performed between On or Off angular distributions and 45°; all On (G): p < 0.001, all Off (G): p = 0.32; red On (F): p < 0.001 (red versus UV), Off: p = 0.24 (red versus UV).

To systematically assess how BC color opponency is generated and/or destroyed by AC circuits, we again allocated BC terminals into spectral groups (Figures 7F and 7G, cf. Figure 5H). Again, there was very little overall change among color-opponent groups, yet more than half of individual terminals that exhibited color opponency under control conditions lost their opponency following AC block (n = 29 of 49, 59.2%). At the same time, an almost equal number of previously non-opponent BCs replenished the population of color-opponent BCs (n = 24). This switching of opponent BCs between conditions affected all three opponent groups; only 2 of 8 (25%), 2 of 7 (29%), and 15 of 33 (45%) long-, mid-, and short-wavelength opponent terminals, respectively, maintained their opponency throughout the pharmacological manipulation. Except for a single BC that switched from long-to short-wavelength opponency, all remaining opponent BCs lost their opponency altogether following AC block. In fact, for all three color-opponent groups, individual examples could be identified where color opponency was either preserved (Figures S8E–S8G), lost (Figures S8H–S8J), or gained (Figures S8K–S8L) following AC block. The overall picture, therefore, is that ACs exert different actions on color opponency in different BC terminals; in some terminals, ACs contribute to generation of color opponency, but in others they masked preexisting opponency. These opposing effects of ACs were exerted on all three color-opponent channels in approximately equal measure.

### ACs modulate BC spectral processing via the on channel

The dominance of color-opponent AC-circuits in the On layer (Figure 3H) suggests that these ACs have a role in determining how the output from BCs is spectrally tuned. To test this idea, we analyzed the color-step responses of the paired dataset, which included many examples of spectrally selective modulation of On signals but spectrally non-selective modulation of Off signals (Figures 7A–7D). For example, under control conditions and following block of inhibition, the Off terminal shown in Figure 7A exhibited spectrally broad Off responses, and ACs acted as achromatic controllers of gain and kinetics. In contrast, the On terminals shown in Figures 7A–7D exhibited notable spectral changes after block of inhibition. This apparent On dominance in AC-dependent spectral re-tuning was particularly striking in the example shown in Figure 7D; this BC terminal exhibited spectrally non-selective Off responses under control conditions, but block of inhibition unmasked spectrally selective short-wavelength On responses (arrowheads), making this terminal color opponent overall.

To systematically assess the role of On and Off circuits for shaping chromatic and achromatic circuit functions, responses to each color step were again fitted with a weighted sum of four kinetic building blocks. We reasoned that any achromatic effects of ACs on BCs should lead to a high degree of covariation across wavelengths, while any spectral “retuning” of BCs should manifest in some wavelength responses being affected more than others. To test this, the degree of response covariation across wavelengths was assessed for each terminal. The plot in Figure 7E shows how blocking inhibition from ACs changed responses in On and Off terminals during red stimulation (dRed) plotted against the corresponding amplitude changes in response to UV stimulation (dUV). The equivalence line at 45° is the case where responses to red and UV co-vary perfectly. Most Off responses (black dots) fell near the equivalence line, but On responses showed a mixture of behaviors, with a second population falling on or near the 0° line (arrowhead) representing response amplitudes changing to UV steps but not red. Only a few points fell on the 90° line, indicating a notable absence of On responses that were modulated in red without also being modulated in UV.

To summarize this behavior, we computed the corresponding angular histogram (Figure 7F), which showed a single peak around 45° for Off responses, indicating mostly co-variation, but two main peaks for On responses: one at 45° and another at 0°. This general pattern was stable for all possible color combinations (Figure 7G; individual color pairs are shown in Figures S10A–S10F). In the On channel, but not in the Off channel, shorter-wavelength responses were modulated more strongly than long-wavelength responses.

This analysis provides further evidence that most spectral modulation of BCs by ACs occurs via the On channel, with short-wavelength circuits being key targets of this modulation.

## Discussion

Investigations of visual processing have usually dealt with stimulus dimensions of space and time separately from color. Here we have shown that grayscale and color processing interact through inhibitory circuits in the inner retina that vary between different zones (Figures 1–3). Blocking inhibition from ACs increased gain in BCs and made responses more transient, as well as unmasking mixed On-Off responsivity that was much more common in the peripheral retina compared with the center (Figures 4 and 5). Simultaneously, ACs contributed to generation of color opponency in the central retina, but in the periphery there was a mixture of effects, enhancing color opponency in some BCs while suppressing pre-existing opponency in others (Figures 3, 5, and 6). ACs counteracting intrinsic color opponency of BCs acted with a high degree of specificity through just one of the two fundamental channels for grayscale processing: the On pathway (Figure 7). We conclude that the central and peripheral retina of larval zebrafish employ fundamentally distinct inhibitory circuits to control the interaction between grayscale and color processing.

### Anisotropic AC-BC circuits

Many vertebrates, including zebrafish and primates, feature a retinal region of peak spatial acuity surrounded by lower-density circuits in the periphery.^48^ Here, spatial acuity is increased by pooling across smaller numbers of cones, but this comes at a cost of reduced signal to noise. In primates^49^ and zebrafish,^34^ this problem is in part addressed by prolonging the integration time of central photoreceptors, providing one possible explanation for the more sustained nature of zebrafish central AC and BC circuits compared with the periphery (Figures 1 and 5).

Next, the cone signal must travel to the RGCs, and any lateral inhibitory circuits on this path are expected to add noise. Correspondingly, primate midget RGCs receive relatively fewer AC inputs compared with their peripheral counterparts,^34^ while larval zebrafish have a reduced central AC-BC ratio compared with the periphery.^33^ Here, our finding that pharmacological removal of AC-mediated inhibition had notably stronger and more diverse effects on BC functions in the periphery compared with the central retina (Figure 5) lends further credence to the idea of a generally reduced inhibitory tone in central circuits.

### The role of ACs in color vision

In larval zebrafish, two forms of color opponency are established at the level of cone outputs,^7^ but three are observed at the level of the downstream BCs.^18^ Accordingly, the expectation is that the “third” form of opponency (UV-yellow) is set up by ACs. This was found to be the case in the central retina (Figures 5H and 5I), but in the periphery, the population representation of color opponency was remarkably invariant to pharmacological removal of inner retinal inhibition (Figure 5H). At the level of individual BCs, however, ACs could either enhance or suppress color opponency. These opposing effects were balanced across BCs by a combination of factors. First, a majority of ACs was essentially achromatic (Figures 3A–3C), indicating that they do not alter spectral processing in a direct manner. Second, a minority of chromatic ACs appear to implement a switch by which they mask pre-existing color opponency in some BCs while at the same time generating qualitatively equivalent information elsewhere (Figures 5J, 6F, and 6G). This switch was implemented mostly by On circuits (Figure 7), and, correspondingly, the dendrites of ACs that exhibit spectral opponency were located in the traditional On layer (Figure 3H).

While it seems intuitive to suggest that color-opponent ACs underpin color processing in BCs, it may also be that non-opponent ACs are involved in the same task. In principle, combining a spectrally broad AC with a spectrally narrow BC would lead to opponency, while combining an intrinsically opponent BC with a spectrally narrow or V-shaped AC might abolish the opponency. Indeed, blocking inhibition from ACs affected BCs’ color-opponent signals across the entire IPL, including in the Off layer, where color-opponent ACs are absent. Notably, similar arguments have been made surrounding the emergence of opponent and non-opponent midget circuits from green- and red-biased LWS (long-wavelength-sensitive) cones in primates;^30^ randomly combining a single such cone in the center with multiple cones in the periphery inevitably leads to opponency. However, at the same time, random combination of multiple such center-surround units downstream will again cancel the opponency to set up a non-opponent luminance pathway.

In the future, the different anatomical distributions of color-coding BCs in the presence and absence of AC inputs (Figure 5J) may provide an important handle for studying the diverse AC-BC circuits that contribute to this overall spectral balancing. Understanding how these correlative observations are causally linked will likely require use of transgenic lines that allow more selective interference with specific types of BCs and ACs. The same strategy should also help to decipher BC circuits where ACs mask a pre-existing opponency.

### A special role of On circuits in zebrafish color vision?

Most AC-mediated spectral tuning in BCs, whether leading to changes in opponency or simply a rebalancing of non-opponent spectral tunings, were predominately implemented via the On rather than the Off channel (Figures 3H, 5J, and 7). This observation adds to a growing body of evidence that zebrafish generally leverage On rather than Off circuits to compute diverse aspects of color information. For example, at the level of the retinal output^33^ and within the brain,^32,50,51^ most spectral diversity is represented in the On channel. In contrast, the spectral tuning function of the brain’s overall Off response essentially resembles the spectral tuning function of red cones in isolation, which also corresponds with the mean spectrum of natural light in the zebrafish natural habitat.^7,32^ From here, it is tempting to speculate that zebrafish generally use the Off channel as an “achromatic reference,” while On circuits can, where required, provide spectrally biased points of comparison to serve spectral and color vision. A predominant use of one rather than both polarities for encoding spectral information could also be advantageous in that it might permit largely unaltered travel of the red cones’ achromatic signal to the brain; by restricting the bulk of spectral computations to the On strata of the IPL, circuits within the Off strata can operate in an essentially achromatic manner. In agreement, the vast majority of ACs in the Off layer exhibited such achromatic tunings (Figures 3A and 3E). In the future, it will be interesting to test whether such an On dominance amongst spectral computations is also a feature in other species.

The possible link between polarity and distinct spectral functions also raises the question how On-Off responses are used for spectral coding. Among ACs, we observed a strong link between color opponency and On-Off responses (Figures 2B and S2). Correspondingly, among BCs, intrinsic On-Off responses were routinely unmasked by blocking ACs, particularly in the central retina, where they were present throughout the On layer (Figures 5A, 5B, 5E, 5F, and S7A). In the future, it will therefore be important to probe more directly to what extent On-Off processing in ACs and BCs can be causally linked to specific aspects of color processing. Another unanswered question is the source of BCs’ On-Off responses. Opponency in cones alone is unlikely to explain this observation. This is because cone inversions from their intrinsic Off response to an HC-mediated overall On response occurs exclusively at long wavelengths;^7^ however, many unmasked On-Off BCs were short wavelength biased (Figure S7A). Instead, as for the observed intrinsic UV-yellow opponent BCs (see above), their existence points to the presence of yet unexplained mechanisms of signal transfer between cones and BCs in the zebrafish retina. One putative mechanism might involve On-acting excitatory amino acid transporters (EAATs).^52,53^

### Color opponency in the absence of ACs

The complex interplay of masked and generated BC opponencies in the absence of inner retinal inhibition confirms that BCs inherit diverse spectral opponencies from the outer retina.^5,7,18^ However, the full picture is decidedly more complex than anticipated from previous work. That long- and mid-wavelength opponencies can be preserved in BCs in the absence of ACs is perhaps expected because these two axes are already fully represented by the two mid-wavelength cones.^7^ However, it remains unclear how the third, short-wavelength opponent axis can persist. While, as with primates,^54^ zebrafish UV cones (SWS1) also exhibit weak but significant UV-yellow opponency,^7^ it seems implausible that this can account for the observed effects. First, this pre-existing outer retinal opponency would need to be substantially boosted to match the much more pronounced opponency in BCs.^18^ Second, in cones, this opponency was restricted to the central retina, and, therefore, it cannot account for the profusion of BCs’ UV-yellow opponencies observed in the periphery.^18^

The continued presence of UV-yellow opponency in BCs following AC block strongly points to the existence of a mechanism capable of selectively inverting cone signals within single BCs. Three potential and non-mutually exclusive mechanisms present themselves. First, a single BC might express depolarizing and hyperpolarizing glutamate receptor systems at the dendritic tips that contact different cones (see also the above discussion on BCs’ On-Off signals). Second, BCs could receive direct inputs from HCs. For example, a putative BC driven by sign-inverted inputs from UV cones (i.e., UV On) could simultaneously receive sign-preserving inputs from H1 and/or H2 HCs, which themselves carry a sign-preserving, long-wavelength-biased signal.^7^ In zebrafish, the presence of direct inputs from HCs to BCs has not been observed; however, the concept is tentatively supported by the anatomical presence of HC-BC contacts in mice.^55^ Third, zebrafish might have ACs that use fast neurotransmitters other than GABA and/or glycine, which presumably continued to function throughout our pharmacological interventions. For example, mice feature VGlut3 ACs, a population of part-glutamatergic ACs implicated in motion processing.^56–58^ Similarly, another key neuron implicated in mammalian motion processing is the starburst AC (SAC), which co-releases acetylcholine alongside GABA.^59,60^ However, the functional role of SACs outside of mammals remains sparsely explored.^61^

### The role of green- and blue-cone circuits in supporting inner retinal color processing

Unlike red and UV cones, zebrafish green and blue cones provide strongly color-opponent outputs because of feedforward signals from the HCs.^7^ Accordingly, these cones might directly support color opponency in BCs. In support of this hypothesis, the spectral zero crossings marked by these two cones remain represented within BCs in the presence and absence of ACs. However, only a minority of green- and blue-cone-like BCs retained their specific opponency upon AC block (Figure 6F). This suggests that, while the signals from green and blue cones can be directly used to support color opponency in BCs, this motif is by no means dominant when considering the complete circuit. Instead, most BC circuits that represent these two spectral opponencies required inputs from ACs. In zebrafish, green but not blue cones provide a cone-type-exclusive drive for at least two anatomically distinct types of BCs,^12^ providing a possible neural substrate for the minority of green-cone-like BCs that were unaffected by AC block. These might account for some of the unmasked green-cone-like BCs when ACs were blocked. Possible green-cone-exclusive BCs might also link with the observation that green light stimulation could result in long-wavelength-biased spectral effects on BCs (Figures S10D and S10E) and that most opponent ACs seemed to be partially built from green cone inputs (Figures S5D and S5E).

In contrast, the possible roles of blue cones in zebrafish color vision remain much more elusive. A blue-cone-exclusive BC is not known to exist,^12^ which leaves the origin of any intrinsic blue-cone-like BC tunings unclear. Further, we found no evidence of any major involvements of blue cones in AC processing (Figures S5D and S5E). On the other hand, the four LEDs used in the present study were not optimally placed to disambiguate blue- from UV-cone contributions (Figures S5B and S5C). Nevertheless, our findings add to a perhaps puzzling body of evidence that questions a key role of blue cones in shaping larval zebrafish vision.^5^

### Balancing color circuits?

As the visual signal travels from the zebrafish outer to the inner retina and then the brain, two perhaps conflicting processing needs arise. (1) Spectral information is already well represented at the level of the cone output,^7^ implying that their spectral signals must be transmitted to the brain without loss.^32^ Our results suggest that ACs solve part of this conflict by rebuilding some of the color-opponent channels that get lost during grayscale processing. (2) With two of four cones displaying strong opponency,^7^ it is perhaps inevitable that many BCs will inherit some form of opponency as they sum across multiple cone types.^5,12,62^ However, for grayscale processing, such color opponency is likely to be detrimental; when an opponent circuit is stimulated with spectrally broad light, the opponency will limit the signal-to-noise ratio or cancel the signal altogether. It therefore makes perhaps intuitive sense that ACs should pre-empt this problem by canceling inherited color opponency in a subset of BCs.

Accordingly, in this view, there are two sides to the same problem: rebuilding of opponency for color processing and cancellation of opponency for grayscale processing. Our finding that the abundance of these two processes is approximately matched suggests that, in effect, ACs “dynamically balance” color and grayscale circuits so that both can coexist. Nevertheless, it remains possible that this balance is merely a by-product of both processes going on in parallel rather than a processing goal in its own right. Here, future studies into how this problem is managed in the retina of other species might provide further insights. Similarly, it will be interesting to explore whether similar solutions have been found in other systems that leverage dense and laterally connecting inhibitory networks.

### An evolutionary perspective

Our results provide tentative insights into the evolution of computation in the brain: in vertebrates and diverse invertebrate eyes alike, the evolution of color computations likely preceded the evolution of complex spatiotemporal vision. This is because (1) opsins, including their immediate spectral diversification, preceded the evolution of highly resolved spatial vision in any animal by some 250 million years;^63^ (2) all extant vertebrates, including lampreys, feature subsets of the same four ancestral opsins across their photoreceptors;^4,5^ and (3) particularly in shallow water, where vision first evolved, spectral information provides a wealth of behaviorally critical cues that do not categorically need supplementation with spatial information (discussed, e.g., in Baden,^5^ Nilsson,^63^ and Maximov^64^). From here, it seems plausible that the earliest forerunners of vertebrate eyes^65^ gradually evolved the bulk of their inner retinal circuits on top of well-functioning outer retinal circuits that already provided useful spectral information. In such a scenario, inner retinal circuit evolution would have occurred under constant selection pressure to maintain coding efficiency for color vision, perhaps explaining the arrangement that we see in zebrafish today. This interpretation would further imply that perhaps also in other layered networks of brains, the primary function of some microcircuits may not be to create new computations but rather to make up for computations that would otherwise be lost.

### Limitations of the study

With a central focus on the encoding of spectral and temporal information, we did not address the encoding of space, an aspect that will be key to investigate in the future. Moreover, to gain a first overview of AC effects on BCs, pharmacological manipulations were necessarily broad, targeting GABAergic and glycinergic circuits alike. Future work aimed at deciphering their presumably distinct contributions to the overall picture will be key, as will be added investigation of the roles of some of the many other transmitter systems used by ACs. With regard to circuits, we here focused on all BCs, enabling their differentiation by stratification alone; however, in the future, targeting specific sub-populations of BCs, alongside their postsynaptic RGC targets, will likely prove to be important for deepening our understanding on inhibitory interaction in the inner retina.

## Star★Methods

## Key Resources Table

**Table:** 

REAGENT or RESOURCE	SOURCE	IDENTIFIER
Chemicals, peptides, and recombinant proteins		
Gabazine	sigmaaldrich	Cat# S106-10MG
TPMPA	sigmaaldrich	Cat# T200-10MG
Strychnine	sigmaaldrich	Cat# S8753-25G
Deposited data		
2-photon imaging data for ACs	This manuscript	https://doi.org/10.5061/dryad.vx0k6djwf http://www.retinal-functomics.net http://www.badenlab.org/resources
2-photon imaging data for paired BCs	This manuscript	https://doi.org/10.5061/dryad.vx0k6djwf http://www.retinal-functomics.net http://www.badenlab.org/resources
2-photon imaging data for population BCs	This manuscript	https://doi.org/10.5061/dryad.vx0k6djwf http://www.retinal-functomics.net http://www.badenlab.org/resources
Experimental models: Organisms/strains		
Danio rerio (zebrafish): *Tg(ptf1a:Gal4)*	Nikolaev et al. (2013)^25^	N/A
Danio rerio (zebrafish): *Tg(UAS:SyGCaMP3.5)*	Nikolaev et al. (2013)^25^	N/A
Danio rerio (zebrafish): *Tg(ribeye:Gal4)*	Rosa et al. (2016)^46^	N/A
Danio rerio (zebrafish): *Tg(ribeye:syGCaMP8m)*	This manuscript	N/A
Software and algorithms		
Igor Pro	Wavematrics	N/A
MATLAB	Mathworks	N/A
ImageJ	https://ImageJ.net/	N/A

## Resource Availability

### Lead contact

Further information and requests for resources and reagents should be directed to and will be fulfilled by the lead contact, Tom Baden (t.baden@sussex.ac.uk).

### Materials availability

All unique reagents generated in this study are available from the lead contact without restriction.

## Experimental Model and Subject Details

### Animals

All procedures were performed in accordance with the UK Animals (Scientific Procedures) act 1986 and approved by the animal welfare committee of the University of Sussex. Animals were housed under a standard 14:10 day/night rhythm and fed three times a day.

Animals were grown in 0.1 μM 1-phenyl-2-thiourea (Sigma, P7629) from 1 *dpf* to prevent melanogenesis. For all experiments, we used 6–8 days post fertilization (*dpf*) zebrafish (Danio rerio) larvae. For 2-photon *in-vivo* imaging, zebrafish larvae were immobilised in 3% low melting point agarose (Fisher Scientific, BP1360-100), placed on a glass coverslip and submerged in fish water. Eye movements were prevented by injection of α-bungarotoxin (1 nL of 2 mg/mL; Tocris, Cat: 2133) into the ocular muscles behind the eye.

### Method Details

#### Light stimulation

With fish mounted on their side with one eye facing upwards toward the objective, light stimulation was delivered as full-field flashes of light. For this, we focused a custom-built stimulator through the objective, fitted with band-pass filtered light-emitting diodes (LEDs) (‘red’ 588 nm, B5B-434-TY, 13.5 cd, 8°; ‘green’ 477 nm, RLS-5B475-S, 3-4cd, 15°, 20 mA; ‘blue’ 415 nm, VL415-5-15, 10–16 mW, 15°, 20 mA; ‘UV’ 365 nm, LED365-06Z, 5.5 mW, 4°, 20 mA; Roithner, Germany). LEDs were filtered and combined using FF01-370/36, T450/pxr, ET420/40 m, T400LP, ET480/40x, H560LPXR (AHF/Chroma). The final spectra approximated the peak spectral sensitivity of zebrafish R-, G-, B-, and UV-opsins (Figures S5B–S5D), respectively, while avoiding the microscope’s two detection bands for GFP and mCherry. To prevent interference of the stimulation light with the optical recording, LEDs were synchronized with the scan retrace at 1 kHz (1 ms line duration) using a microcontroller and custom scripts. Further information on the stimulator, including all files and detailed build instructions can be found in ref. ^43^.

Stimulator intensity was calibrated to be spectrally flat at 30 mW per LED, which corresponds to low-photopic conditions. Owing to 2-photon excitation of photopigments, an additional constant background illumination of ~10^4^ R* was present throughout.^66,67^ For all experiments, larvae were kept at constant illumination for at least 5 s after the laser scanning started before light stimuli were presented. Three types of full-field stimuli were used: (i) a spectrally flat ‘white’ chirp stimulus^68^ where all four LEDs were driven together, (ii) flashes of light at each of the four wavelengths (2 s On, 2 s Off), and (iii) a binary dense and spectrally flat white noise, in which the four LEDs were flickered independently in a known random binary sequence at 6.4 Hz for 300 s.

#### 2-Photon calcium imaging

All 2-photon (2P) imaging was performed on custom-built 2P microscope equipped with a mode-locked Ti:Sapphire laser (Chameleon 2, Coherent) tuned to 915 nm for SyGCaMP imaging. Emitted photons were collected through the objective (Nikon, MRD77225, 25X) as well as through an oil condenser (NA 1.4, Olympus) below the sample using GaAsP photodetectors (H10770PA-40, Hamamatsu). For image acquisition, we used ScanImage software (r 3.8) running under MATLAB (R 2013b). All recordings were taken at 128 × 100 pixels (10 Hz frame rate at 1 ms per scan line).

#### Pharmacological manipulation

For pharmacological AC blockage, we injected ~4 nL of a solution containing antagonist to GABA_A_, GABA_C_ and glycine receptors into the anterior chamber of the retina. The estimated final concentration in the extracellular space was 5 μM gabazine (Sigma) as antagonist of GABA_A_ receptors; 5 μM TPMPA (Sigma) as antagonist of GABA_C_ receptors; 5 mM strychnine (Sigma) as antagonist of glycine receptors.^26^ The solution also contained 1 mM Alexa 594 for verifying the quality of the injection.

#### Data analysis

Data analysis was performed using IGOR Pro 6.3 and 8.2 (Wavemetrics), Fiji (NIH) and MATLAB R2019b/R2020b (Mathworks).

#### ROI placement, IPL detection and functional data pre-processing

Where necessary, images were xy-registered using the registration function provided in SARFIA^69^ running under IGOR Pro 8.2. For AC data, ROIs were defined automatically based on local image correlation over time, as shown previously.^13^ For densely labeled SyjGCaMP8m BC data, regions of interests (ROIs) were placed automatically after local thresholding of the recording stack’s standard deviation (typically >20) projection using the tetrachromatic noise data, followed by filtering for size and shape using custom-written scripts running under IGOR Pro 8.2 (WaveMetrics), as described previously.^34^ For sparsely labeled SyGCaMP3.5 BC data, ROIs were drawn by hand based on the standard deviation projection across the tetrachromatic noise data. The ROIs from control condition and drug condition were drawn separately. Terminals were paired across the two conditions using the experimenter’s best judgment, which we found to be more reliable than automated procedures. The matching of terminals across conditions was greatly facilitated by the sparse expression strategy, and throughout we tried to be as conservative as possible to only match terminals when we were certain that they are the same ones (i.e. minimising false positives, at the expense of false negatives). For cone recordings, the ROIs were drawn manually from the standard deviation projection across time.

In all scans within the IPL layer, IPL boundaries were drawn by hand using the custom tracing tools provided in SARFIA.^69^ The IPL positions were then determined based on the relative distance of a ROIs’ center of mass between the IPL boundaries and mapped to the range 0%–100%.

Fluorescence traces for each ROI were z-normalised, using the time interval 2–6 s at the beginning of recordings as baseline. A stimulus time marker embedded in the recording data served to align the Ca^2+^ traces relative to the visual stimulus with a temporal precision of 1 ms. Responses to the chirp and step stimuli were up sampled to 1 kHz and averaged over 5 trials. For data from tetrachromatic noise stimulation we mapped linear receptive fields of each ROI by computing the Ca^2+^ transient-triggered-average. To this end, we resampled the time-derivative of each trace to match the stimulus-alignment rate of 500 Hz and used thresholding above 0.7 standard deviations relative to the baseline noise to the times *t_i_* at which Calcium transients occurred. We then computed the Ca^2+^ transient-triggered average stimulus, weighting each sample by the steepness of the transient:

$$F(l,\tau )=\frac{1}{M}\sum_{i=1}^{M}\dot{c}\left(t_{i}\right)S\left(o,t_{i}+\tau \right)$$

Here, ***S***(*l,t*) is the stimulus (“LED” and “time”), τ is the time lag (ranging from approx. -1,000 to 350 ms) and *M* is the number of Ca^2+^ events. The resulting kernels are shown in z-scores for each LED, normalised to the first 50 ms of the time-lag. To select ROIs with a non-random temporal kernel, we used all ROIs that exceeded a standard deviation of ten in at least one of the four spectral kernels. The precise choice of this quality criterion had no major effect on the results.

#### Kernel polarity

The use of a fluorescence-response-triggered average stimulus (here: ‘kernel’) as a shorthand for a neuron’s stimulus-response properties, while potentially powerful (e.g. Ref. ^13,19 and 33^), ought to be considered with some caution. For example, determining a binary value for a kernel’s polarity (On or Off) can be conflicted with the fact that a neuron might exhibit both On and Off response aspects. Moreover, different possible measures of On or Off dominance in a kernel can generate different classification biases. Here, following our previously established approach^19,33^ we defined On and Off based on a measure of a kernel’s dominant trajectory in time. Before the calculation, we first smoothed the kernels to eliminate the high-frequency noise. After that, we determined the position in time of each kernel’s maximum and minimum. If the maximum preceded the minimum, the kernel was classified as Off, while vice versa if the minimum preceded the maximum, the kernel was defined as On.

#### Reconstruction of step responses using kinetic components

To reconstruct each cell’s mean response into constituent spectral and temporal components we used four temporal components associated with a given light response (i.e. 3 s light, 3 s dark for ‘white’ steps, and 2 s light 2 s dark for ‘color’ steps), following a previously described approach.^18^ The temporal components used resembled light-transient, light-sustained, dark-transient, and dark-sustained temporal profiles (Figure S1B). These components were fitted to the trial-averaged step responses of individual ROIs in sequence, in each case minimising the mean squared difference between a template’s peak and the corresponding five time-points in measured response, with previously fitted components subtracted. The fit sequence was: Light-sustained, light-transient, dark-sustained, dark-transient. This yielded four corresponding weights, scaled in z-scores in accordance with the amplitudes of the trial averaged response means.

To assess reconstruction quality, reconstructed data was subtracted from the original ROI-means to yield residuals. From here, we compared original data, reconstructions, and residuals based on variance explained across all ROIs (as in ref. ^18^). To this end, we first computed the total variance across all clusters for each time point, computed the area under the curve for each variance-trace, and normalised each to the result from the original cluster means. By this metric, reconstructions captured 98%, 97%, 95% and 96% of the total variance for the ‘white-control’, ‘white-AC-block’ and ‘color-control’ and ‘color-AC-block’ steps, respectively.

Response polarity per ROI was then computed as follows. A ROI was considered as displaying an On-response if the sum of the light-transient and light-sustained weights exceeded 3 SD. A ROI was considered as displaying an Off-response if either the sum of the light-transient and light-sustained weights was more negative than −3 SD, or if the sum of the dark-transient and dark-sustained components exceeded 3 SD. If by these criteria a ROI display both On- and Off-responses, it was counted as On-Off. ROIs failing to elicit either On- or Off-responses were counted as non-responsive.

#### Clustering of ACs

Clustering was performed on the dataset containing the functional responses of ACs to chirps, color steps and kernels derived from the color noise stimulus. All input traces were up sampled to 1 kHz (cf. pre-processing) which yielded n = 25,000 points (chirp), four times n = 4,000 points (steps) and four times n = 1,299 points (kernels). Responses to all three stimuli were used for the clustering.

Regions of interest (ROIs) with low-quality responses to all three stimuli were identified and removed from the dataset, ROIs with a high-quality response to at least one stimulus being retained in all cases. The quality of response to the chirp and step stimuli was determined using the signal-to-noise ratio quality index: *QI* = *Var*[⟨***C***⟩_*r*_]_*t*_ /⟨*Var*[***C***]_*t*_⟩_*r*_, where *C* is the *T* by *R* response matrix (time samples by stimulus repetitions), and ⟨·⟩_*x*_ and *Var*[·]_*x*_ denote the mean and variance respectively across the indicated dimension, *x* ∈ {*r, t*} (see ref. ^68^). A quality threshold of 0.35 was chosen, below which responses were judged to be of poor quality. We calculated the standard deviation in the light intensity over time for each stimulus color in the kernel (R, G, B and UV). The kernel quality of each ROI was defined as the maximum standard deviation across the four colors. A kernel quality threshold of 5 was chosen, below which kernels were judged to be of poor quality. The raw dataset was of size n = 1,776. Following quality control, the dataset was of size: n = 1,743 (98.1% of the original).

We scaled the data corresponding to each chirp, step color and kernel color by dividing each one by the standard deviation through time and across ROIs. In this way we ensured an even weighting between stimuli.

We used principal component analysis (PCA) to reduce the dimensions of the problem prior to clustering. PCA was performed using the MATLAB routine pca (default settings). We applied PCA separately to the chirps and to the portions of a dataset corresponding to each of the step and kernel colors, retaining the minimum number of principal components necessary to explain ≥99% of the variance. The resulting nine ‘scores’ matrices were then concatenated into a single matrix ready for clustering. The following numbers of principal components were used – chirp: 41; step: 8 R components, 9 G components, 13 B components and 13 UV components; kernels: 7 R components, 16 G components, 31 B components and 21 UV components, giving 159 PCA components in total.

We clustered the combined ‘scores’ matrix using Gaussian Mixture Model (GMM) clustering, performed using the MATLAB routine **fitgmdist**. We clustered the data into clusters of sizes 1,2,?,50, using i) shared-diagonal, ii) unshared-diagonal, iii) shared-full and iv) unshared-full covariance matrices, such that (50*4 =) 200 different clustering options were explored in total. For each clustering option 20 replicates were calculated (each with a different set of initial values) and the replicate with the largest log likelihood chosen. A regularisation value of 10^-5^ was chosen to ensure that the estimated covariance matrices were positive definite, while the maximum number of iterations was set at 10^4^. All other **fitgmdist** settings were set to their default values.

The optimum clustering was judged to be that which minimised the Bayesian information criterion (BIC), which balances the explanatory power of the model (log likelihood) with model complexity (number of parameters). Lastly, clusters with <10 members were removed.

Using the above procedure, we obtained 23 clusters (1 cluster with <10 members was removed), with unshared diagonal covariance matrices providing the optimal solution. Finally, we split n = 4 clusters with a notably bimodal IPL distribution into their On- and Off-stratifying components (IPL-cut at 60% depth), yielding a total of 27 response groups. To what extent the 27 clusters correspond to AC-types remains unknown, and in view of >60 AC types in mice,^22^ 27 putative types in zebrafish probably underestimates their full diversity, which is likely part related to the necessarily incomplete sampling of the full stimulus space and/or possible incomplete labeling of the ptf1a driver.

#### Fitting of AC-cluster spectral tuning functions with cones

Spectral tuning functions of AC clusters means were matched to those of previously recorded cones (cf. Figures S5B and S5C) based on the four relative kernel amplitudes (as shown in Figures 3A–3D). Fitting was done as follows: For each tested cone-combination (e.g. all cones, or R + U only, etc.) we computed 10^6^ possible combined tunings at random by summing the respective reduced cone tuning functions (Figure S5C) with random scaling between −1 and 1 each. We then computed the linear correlation coefficient between each AC-cluster’s tuning function, and each of the randomly generated combined cone-tunings, in each case choosing conecombination that gave the maximal correlation as the best fit. Finally, for each best fit, we scaled all cone weights to minimise the residual between the corresponding AC-cluster’s tuning function and that of the combined cone-tuning. Based on this strategy, the mean variance explained for cone-combinations RGBU, RU, RGU, GU, respectively, were: All long-On/Off clusters: 95.4%, 86.3%, 90.0%, 53.3%; All V-shaped clusters: 84.0%, 81.4%, 84.9%, 55.6%; All opponent clusters: 86.8%, 63.2%, 83.1%, 71.8%.

#### Sorting ACs and BCs into spectral groups

Using the amplitudes of the four kernels, cluster means of ACs were sorted into one of the four spectral groups. First, the AC clusters were divided into non-opponent (Figures 3A–3C) and opponent groups (Figure 3D). Next, For the non-opponent groups, the AC clusters were sorted into the three following groups: Long-Off, with the long-biased Off-response across all four spectral stimuli if *abs*(R + G)>*abs*(B + U)*2; Long-On, as Long-Off but for On-responses; V-shaped: *abs*(R + U)>*abs*(G + B)*2. The opponent group was further sorted into RG/BU (C_21,22_) and RBU/G (C_13,18,23,24_) sub-groups based on the relative signs of green vs. UV-responses.

Kernel amplitudes of each BC terminals were sorted into 8 spectral groups. First, all BCs that failed to exceed a minimum absolute amplitude of 10 SD in at least one of the four kernels was counted as non-responsive. Next, we divided the remaining BCs into non- opponent (groups 1–5) and opponent types (groups 6–8) based on the relative signs of all four (R, G, B, U) LED kernels. From here, the two sets of BCs were sorted further as follows: *Non-opponent BCs*, in order: Long-biased if *abs*(R + G)>*abs*(B + U)*2, Short-biased if *abs*(R + G)<*abs*(B + U), Mid-biased if *abs*(G + B)>*abs*(R + U)*2, V-shaped if *abs*(R + U)>*abs*(G + B)*2, else: “Broad”. Opponent BCs; Short-opponent if (R + G > 0 && U < 0) || (R + G < 0 && U > 0), Mid-opponent if (R > 0 && B < 0) ||R < 0 && B > 0), Long-opponent if (R > 0 && G < 0) || (R < 0 && G > 0). Finally, in rare cases where (R > 0 && G < 0 && U > 0) || (R < 0 && G > 0 && U < 0), BCs were allocated as long-opponent if *abs*(R)>*abs*(U) but as short-opponent *abs*(R)<*abs*(U).

Note that despite the similar sorting procedure, ACs only fell into 4 groups (Figures 3A–3D) while BCs can be sorted into 8 (Figure 5H).

#### Response synchronisation

To determine the degree of response synchronisation within each scan, we used the synchrony measurement method described in ref. ^70^, using the z-normalized fluorescence traces from tetrachromatic noise stimulation of the wide-expressed syGCaMP8m BC data as the input. We first evaluated *F(t)* across all the recorded terminals within one field of view at a given time *t*:

$$F(t)=\frac{1}{N}\sum_{i=1}^{N}F_{i}(t).$$

The variance of the time fluctuations of *F(t)* is

$$\sigma_{F}^{2}={\langle {\left[F\left(t\right)\right]}^{2}\rangle }_{t}-{\left[{\langle F\left(t\right)\rangle }_{t}\right]}^{2}$$

Where ⟨…⟩_*t*_ denotes the time-averaging over the session. For each terminal *F_i_(t)*, we used similar approach to calculate the time fluctuations

$$\sigma_{Fi}^{2}={\langle {\left[F_{i}\left(t\right)\right]}^{2}\rangle }_{t}-{\left[{\langle F_{i}\left(t\right)\rangle }_{t}\right]}^{2}$$

The synchrony measure, χ(*N*), for the scan filed is then calculated as

$$\chi (N)=\sqrt{\frac{\sigma_{F}^{2}}{\frac{1}{N}\sum_{i=1}^{N}\sigma_{F_{i}}^{2}}}$$

The value of χ(*N*) ranges between 0 and 1. χ(*N*) = 1 indicates that all ROIs within a scan are perfectly synchronized.

#### Analysis of cone recordings

To control for possible effects of our pharmacological manipulation on outer retinal processing, we recorded calcium responses from the synaptic output pedicles of the four types of cones as described previously^7^ under control conditions and again following application of the GABA/glycine blocker cocktail, each time assessing cones’ responses to the spectral steps stimulus (Figures S6C– S6N). For each cone, response amplitudes per step were extracted as described previously,^7^ and tuning curves were computed by sign-inverting response amplitudes (to compensate for the hyperpolarising light response of vertebrate cones) and normalising each curve to a peak of 1.

## Quantification and Statistical Analysis

### Statistics

No statistical methods were used to predetermine sample size. Owing to the exploratory nature of our study, we did not use randomization or blinding.

Chi-squared tests were used for the following datasets: polarity and kinetics based on white step stimulation for ACs (Figure 1G); BCs polarity distribution before and after disinhibition with white steps (Figures 5A and 5B); BC response types before and after disinhibition (Figures 5E and 5F, bar plots); BCs polarity distribution before and after disinhibition with color steps (Figure S7); Polarity distribution for paired BCs (Figure 6E); paired BCs polarity distribution before and after sham injection with white steps (Figure S9H).

Wilcoxon signed-rank tests were used for the following datasets: comparison of population synchronicity (Figure S6B); weight distributions for the four kinetic components (Figure 5D); population synchronicity in sham dataset (Figure S9I); co-variation of absolute response amplitudes changes (Figures 7F and 7G and S10A–S10F).

Wilcoxon rank sum tests were used for the following datasets: distribution of kinetic component weights for ACs (Figure 1H); drug effect for four types of cones (Figures S6E, S6H, S6K, and S6N); Distribution of kinetic component weights for BCs (Figures 5E and 5F, distributions); Percentages of ROIs per spectral category (Figure 5H).

Two sample Kolmogorov–Smirnov tests were used for the following datasets: Distribution of On and Off BC-terminals across the IPL based on white steps (Figures 5C and 5D); Distribution of opponent terminals across the IPL based on spectral kernels (Figures 5I and 5J).

## Data Availability

Pre-processed functional 2-photon imaging data and associated summary statistics is freely available on Data Dryad (https://doi.org/10.5061/dryad.vx0k6djwf) and via the relevant links on http://www.badenlab.org/resources, http://www.retinal-functomics. net and https://doi.org/10.5061/dryad.vx0k6djwf. This paper does not report original code. Any additional information required to reanalyze the data reported in this work paper is available from the lead contact upon request.

## In brief

Wang et al. reveal how zebrafish retinal amacrine cells simultaneously destroy and rebuild color information in bipolar cells so that the net change is essentially zero. The authors posit that the role of some inhibitory networks is not to sharpen neural representation but to rebuild information that would otherwise be lost.

## Highlights

- Zebrafish bipolar cells inherit color-opponent signals from the outer retina
- ACs destroy this opponency in some BCs but simultaneously rebuild it in others
- On balance, ACs do therefore not notably alter color processing in BCs
- Some ACs therefore preserve incoming information that would otherwise be lost
